# Regional and seasonal activity predictions for fall armyworm in Australia

**DOI:** 10.1016/j.cris.2021.100010

**Published:** 2021-01-23

**Authors:** James L. Maino, Rafael Schouten, Kathy Overton, Roger Day, Sunday Ekesi, Bosibori Bett, Madeleine Barton, Peter C. Gregg, Paul A. Umina, Olivia L. Reynolds

**Affiliations:** a Cesar Australia, 293 Royal Parade, Parkville, Melbourne, Victoria 3052, Australia; b CABI, Wallingford, UK; c International Centre of Insect Physiology and Ecology, Nairobi, Kenya; d Plant Health Australia, ACT 2600, Australia; e School of Environmental & Rural Science, University of New England, Armidale NSW 2351, Australia; f School of BioSciences, The University of Melbourne, Victoria 3010, Australia; g Graham Centre for Agricultural Innovation, Wagga Wagga NSW 2650, Australia

**Keywords:** Fall armyworm, Dispersal, Model, Population dynamics, Agricultural pest, Invasive species

## Abstract

Since 2016, the fall armyworm (FAW), *Spodoptera frugiperda*, has undergone a significant range expansion from its native range in the Americas, to continental Africa, Asia, and in February 2020, mainland Australia. The large dispersal potential of FAW adults, wide host range of immature feeding stages, and unique environmental conditions in its invasive range creates large uncertainties in the expected impact on Australian plant production industries. Here, using a spatial model of population growth and spread potential informed by existing biological and climatic data, we simulate seasonal population activity potential of FAW, with a focus on Australia's grain production regions. Our results show that, in Australia, the large spread potential of FAW will allow it to exploit temporarily favourable conditions for population growth across highly variable climatic conditions. It is estimated that FAW populations would be present in a wide range of grain growing regions at certain times of year, but importantly, the expected seasonal activity will vary markedly between regions and years depending on climatic conditions. The window of activity for FAW will be longer for growing regions further north, with some regions possessing conditions conducive to year-round population survival. Seasonal migrations from this permanent range into southern regions, where large areas of annual grain crops are grown annually, are predicted to commence from October, i.e. spring, with populations subsequently building up into summer. The early stage of the FAW incursion into Australia means our predictions of seasonal activity potential will need to be refined as more Australian-specific information is accumulated. This study has contributed to our early understanding of FAW movement and population dynamics in Australia. Importantly, the models established here provide a useful framework that will be available to other countries should FAW invade in the future. To increase the robustness of our model, field sampling to identify conditions under which population growth occurs, and the location of source populations for migration events is required. This will enable accurate forecasting and early warning to farmers, which should improve pest monitoring and control programs of FAW.

## Highlights

• We develop a spatial model of population growth and dispersal for the fall armyworm.
• The model accurately predicts the native and invasive ranges across the world.
• Populations could permanently persist in north-eastern Australia where susceptible crops are grown.
• Adults that disperse into cooler regions may be constrained by winter temperatures and aridity.
• Model outputs can directly inform management strategies for the pest moving forward.

## 1 Introduction

The fall armyworm ‘FAW’, *Spodoptera frugiperda* (J. E. Smith) (Lepidoptera: Noctuidae), is native to the tropical and subtropical regions of the Americas, where it is a sporadic pest (Bodkin 1913; Luginbill 1928; Sparks 1979). Since 2016, its global distribution has undergone a large range expansion into the continents of Africa, Asia, the Pacific and, in February 2020, Australia. Fall Armyworm is highly polyphagous, reportedly attacking over 350 commercial and non-commercial hosts (Montezano et al., 2018). Plant species that are particularly prone to attack are those from the Poaceae family, including corn/maize (*Zea mays* L.), sorghum (*Sorghum* spp.), rice (*Oryza sativa* L.) and various pasture grass species, as well as other non-grass crops including cotton (*Gossypium hirsutum* L.) (Casmuz et al., 2010; Montezano et al., 2018). The permanent range of FAW is restricted to warmer climates, but it has significant capacity for migration and annually invades less suitable climatic regions during warmer periods (Nagoshi et al., 2012).

Seasonal and regional activity of plant pests is a key concern in managing risks to food production. For many highly mobile and seasonal pests, knowledge of the climatic responses of population growth and dispersal processes are important components in understanding their ability to exploit transient resources, such as annually grown crops. Therefore, establishing seasonal and spatial relationships in the population dynamics and migratory behaviour of FAW will be key in predicting and mitigating its impact to Australia's vulnerable plant production industries. Given its native and sporadic pest status in the Americas, to date, most research on the seasonal dynamics and migration potential of FAW has occurred in the USA.

Barfield et al. (1980) proposed three hypotheses for the seasonal population dynamics of FAW in North America: (1) long-distance spread from areas that support permanent, year-round populations, (2) spread from local over-wintering refugia, and (3) a combination of the two. Accumulated evidence on the cold susceptibility of FAW suggests that Barfield's second and third hypotheses are unlikely (Foster and Cherry 1987; Ali et al., 1990). For example, when field cages were stocked with larvae and pupae across Florida, Georgia and South Carolina, Luginbill (1928) found that FAW could only overwinter in the warmest, southernmost field site at Wauchula in Florida, which continues to be supported by more recent observations of year-round population activity (Nagoshi and Meagher 2008). In contrast, studies on the long-distance dispersal capacity of FAW support Barfield's first hypothesis. Pair et al. (1991) provided some of the first evidence that large local populations could migrate long distances and cause outbreaks at locations far removed from source populations (e.g. from southern Texas to Missouri, USA). Further, a mixed population of *Helicoverpa zea* (Boddie 1850) and FAW moths, emerging from 200 000 ha of infested maize in the lower Rio Grande Valley, was tracked by airborne radar for almost 8 h (Wolf et al., 1990). They found the moths moved over 400 km downwind (towards the north), flying at heights of 200–700 m and speeds of 12–25 ms^−1^, utilising a low-level jet wind at the migration altitude for a portion of the time.

In Australia, other grass-feeding noctuids, particularly the armyworms *Mythimna convecta* (Walker 1857), *Persectania ewingii* (Westwood 1839) and *Persectania dyscrita* (Common 1954), and the native budworm *Helicoverpa punctigera* (Wallengren) are strongly adapted to breeding in native grasses, and other inland hosts, both within and well beyond grain cropping zones (Farrow and McDonald 1987; Gregg et al., 1993, 1995, 2001). Given the wide host range of FAW, it is likely it too will be able to exploit such niches. However, unlike North America, where seasonality is characterised by annual cold winter cycles, outbreaks of many arthropod pests in Australia tend to be driven by sporadic rainfall events (Farrow and McDonald 1987). Outbreaks of *H. punctigera* in inland regions are strongly associated with above-average autumn and winter rainfall which favours the growth of annual host plants (Farrow and Daly 1987; Gregg et al., 2019). Major outbreaks of other endemic noctuid caterpillars have followed widespread, drought-breaking rains inland, observed in 1907, 1931, 1936, 1947, 1954, 1973 and 1983 (Farrow and McDonald 1987). While the mechanism remains unclear, droughts tend to have negative impacts on natural enemy populations that would otherwise suppress pest populations following rainfall events (Bodkin 1913). However, there are also exceptions to this tendency, with outbreaks of *M. convecta* in Queensland in 1931, 1938, 1948, 1952 and 1978 not associated with any marked annual rainfall anomalies (Farrow and McDonald 1987).

The seasonal activity potential of FAW in Australia remains unstudied, however there have been several attempts to model its global distribution potential, which includes Australia. Early et al. (2018) used an ensemble species distribution modelling approach to predict the environmental niche of year-round permanent populations by excluding records of transient migrating populations (such as those in the majority of North America). Similarly, a CLIMEX species distribution model was developed by du Plessis (2018), which aimed to predict the global distribution of year-round FAW populations. While useful for predicting FAW establishment potential, these studies were limited in their ability to estimate seasonal activity potential of FAW. To make predictions that can be used to guide mitigation of any potential crop damage, four aspects of these modelling approaches must be addressed. Firstly, models should explicitly account for dispersal processes which drive large portions of the observed distribution of FAW (e.g. North America), including outbreaks. Secondly, instead of using dimensionless habitat suitability indices (of between 0 and 1), models should directly estimate population growth at a given location. This allows researchers to better infer how climate might constrain species survival. Thirdly, the temporal resolution of climate suitability should be sufficient such that seasonal patterns can be identified. Finally, interannual variability in regional climatic conditions should be considered, to account for variation in FAW population dynamics between years.

Here, we address these limitations, utilising a predictive model to produce estimates of the activity potential of FAW across Australia through time, by analysing seasonal and regional variability in climate, population growth and dispersal processes for FAW. Specifically, we show how the seasonal distribution of FAW populations in Australia varies throughout a year and between years. This information can be used to guide farmers’ and authorities’ preparedness and management plans as they learn how to manage this new pest. To increase confidence in predictions for Australia, we validated our model against available datasets on seasonal activity in North America.

## 2 Methods

Below we detail the structure and parameterisation of a spatially explicit model of FAW population growth and dispersal that operates at weekly timesteps and a 9-km (81 km^2^) grid cell resolution across the world. Dynamic model processes were captured by several discrete modules: 1) climate-based population growth rate; 2) short-range dispersal; 3) long-range dispersal, and 4) Allee effects (density dependence).

### 2.1 Climate and population growth potential

Population growth potential through time represents the boundary constraints on permanent establishment, rates of spread, and subsequent impacts of FAW. Understanding population growth is important as regional and seasonal variation in ecoclimatic conditions and suitability will cause populations to grow and shrink at different rates. Recent studies also support the idea that the dynamics of FAW are more influenced by the prevailing climatic conditions rather than the number of commercial hosts available, such as maize (Caniço et al., 2020). Moreover, the potential spread of FAW to transient (seasonal) populations will first be dictated by the climatic conditions encountered at the source population. Thus, we firstly developed a population growth model for FAW based on the effects of climate on local population dynamics.

The intrinsic rate of population growth ($r_{m}$) is the exponential growth rate of a stable population (N) through time (t), or $\frac{dN}{dt}=r_{m}N$. Sousa et al. (2016) measured intrinsic population growth rates of different populations of FAW, which were reared on maize. They found the intrinsic rate of increase (*r_m_*) varied between 0.12 and 0.20 at 27 °C, depending on the provenance of the population. This translates to a 29 to 270-fold increase in population size after 4 weeks but represents a large degree of uncertainty on growth potential between populations.

The temperature response of positive growth rate ($r_{p}$) was modelled using a formulation of the Sharpe and DeMichele model for the reaction kinetics of poikilotherm development (Schoolfield et al., 1981), and was parameterised using measured temperature response parameters (Barfield et al., 1978). The fitted non-linear temperature response of development rate (Supp. Fig. S1) shows the development rate of FAW reaches a maximum at 33.5 °C, but thereafter decreases until it is only 35% of its maximum at 40 °C. This model, which was parameterised under constant temperature in laboratory conditions, was then validated under fluctuating thermal regimes that used many temperatures in the extreme range for this species. Fluctuating temperature regimes do not appear to cause large differences in the development of FAW when compared with constant regimes held at the mean of the fluctuating regime (Simmons 1993). Because of this we could be confident these laboratory-measured traits translate relatively well into fluctuating thermal conditions in the field. Ecological characteristics of different FAW strains could conceivably affect ecoclimatic responses (as is documented for other traits, such as pesticide sensitivity (Huang et al., 2014) and host-preference (Meagher and Nagoshi 2004)). However, due to a lack of ecophysiological data for different strains, we were limited to considering ecoclimatic responses of FAW more broadly and ignore potential strain variation in development rates.

To capture periods of population decline, negative growth rate ($r_{n}$) was parameterised from studies of FAW mortality under stress, which is assumed to occur once an environmental variable (s) exceeds some threshold (e.g. critical thermal maximum), beyond which the mortality rate scales approximately linearly with the depth of the stressor (Enriquez and Colinet 2017). Stressor induced mortality can be incorporated through quantifying the threshold ($s_{c}$) beyond which stress associated mortality commences, and the mortality rate parameter ($m_{s}$) which reflects the per capita mortality per stress unit per time. The mortality rate for each stressor (s) can thus be incorporated as $\frac{dN}{dt}=\left(r_{p}-r_{n}\right)N$, where $r_{n}=\;\sum_{s}f\left(s,s_{c}\right)m_{s}$ and $f(s,s_{c})$ is a function that provides the positive units by which s exceeds $s_{c}$. When the intrinsic growth rate is positive, a carrying capacity (K) can be used to place an upper bound on population growth using the simple logistic formulation of $\frac{dN}{dt}=\left(1-N/K\right)\left(r_{p}-r_{n}\right)N$.

Here we consider the thermal stressors (critical maxima and minima) as well as moisture stress (desiccation), which are represented by air temperature, and soil water content. Once these thresholds have been exceeded, the mortality rate ($m_{s}$) for each stressor (s) can be estimated from previous studies using the solution to the intrinsic growth differential equation when growth rate is non-positive, $p=e^{-m_{s}a_{s}t}$ where p is the surviving proportion and $a_{s}$ is the accumulated stress units until time (t).

Temperature stressors are more widely studied than water-mediated stressors among invertebrates, and FAW is no exception. A study on the variable temperature responses for each FAW life stage from egg to pupae showed that lower thresholds for development were similar across all life stages (Ali et al., 1990), averaged at 12.95 °C. At cooler rearing temperatures, larval survival decreased from 90% to 40% as temperatures decreased from 21 °C to 17 °C (Ali et al., 1990). Survival also further decreased when the host plant was changed from maize to cotton. With respect to chronic exposures to cold temperature extremes, Foster and Cherry (1987) measured the survival of cool acclimated FAW of all life stages to 3-h cold exposures at temperatures ranging from 0 °C to −10 °C. Although some mortality was observed at the mildest temperature (0 °C), the majority of all life stages did not survive the 3-h exposure at −10 °C, with cold tolerance generally decreasing for older life stages (Supp. Fig. S2). These measurements of mortality and development rates at different temperatures were substituted into the non-positive growth rate equation to solve the mortality rate value ($m_{s}$). For example, $p_{T_{\text{min}}}=e^{-m_{T_{\text{min}}}\;\left(-12.97\;-\;5\right)\left(3/24\right)}=64\%$

In contrast to lower temperature thresholds for development, there has been less research conducted on the upper temperature thresholds for development of FAW. Larval survival decreased from 75% to 55% as rearing temperature increased from 33 °C to 35.5 °C when reared on maize (Ali et al., 1990). A polynomial function was fitted to survival data, and estimated a maximum viable temperature of 39.8 °C (Valdez-Torres et al., 2012). However, larval development did not occur when reared at a constant temperature of 37.8 °C (Barfield et al., 1978) or at 38 °C (Ali et al., 1990). Thus, we assumed an upper threshold as 39.8 °C, as other studies have done (Du Plessis et al., 2018), with a mortality rate that leads to 10% daily mortality after exposure to 45 °C. Consequently, we can again solve for the mortality rate as $p_{T_{max\;}}\;=e^{-m_{T_{max\;}}\left(\;45\;-\;39.8\right)\left(1\right)}=90\%$

High moisture availability can cause stress in invertebrates; however, we have not considered high moisture stress here. Previous studies on FAW have found that the level of humidity present does not appear to be a critical factor influencing the length of the egg or pupal stages (Luginbill 1928), or pupal deformity incidence (Simmons 1993), with only small effects observed on the weight at eclosion (Simmons 1993). However, a single field study showed that simulated rainfall of 0, 20 and 80 mm caused increasingly reduced pupal emergence, possibly due to pre-emergent moths being trapped in pupal tunnels by loose soil (Sims 2008), though in other pests higher moisture can also result in higher mortality through fungal pathogens (Ekesi et al., 2003).

Stress through low water availability can cause direct impacts on FAW populations due to desiccation, but also indirectly through effects on vegetation and host availability (Farrow and McDonald 1987). Indirect effects of moisture are likely to be more important for FAW population sizes than the previously discussed direct effects. Just as cold winters may reduce natural enemy populations leading to outbreaks of FAW (Luginbill 1928), periods of drought followed by rain may be readily exploited by FAW due to the sudden availability of hosts and lack of natural enemies, which are often less mobile and less capable of colonising newly suitable localities (Bodkin 1913; Silvain and Ti-A-Hing 1985; Farrow and McDonald 1987). Water (and food) availability during the adult stage appears crucial for oviposition to occur (Luginbill 1928). Unfed moths were found to seldom, if ever, oviposit, and apparently do not mate, though, well-fed females may also occasionally die without ovipositing (Luginbill 1928). Thus, we assumed desiccation stress to occur once 50% of the soil in a grid cell is at wilting point, representing 10% daily mortality when soil is fully dry and solve the mortality rate as $p_{dry}=e^{-m_{wilting}\;\left(\;0.5-0.0\right)\left(1\right)}=90\%.$

Estimates for threshold parameters of climatic stressors are shown in Table 1 with supporting empirical datasets (and, where necessary, extrapolated performance curves provided in Supp. Fig. S1 and Fig. S2).

**Table 1:** Parameters for critical thresholds and mortality rates for key environmental stressors.

Parameter	Description	Value	Justification
$C_{T_{\min}}$	Critical minimum temperature, °C	12.97	(Ali et al., 1990)
${m_{T}}_{\min}$	Mortality rate per cold stress, °C/d	0.2	(Foster and Cherry 1987)
$C_{T_{\max}}$	Critical maximum temperature, °C	39.8	(Valdez-Torres et al., 2012; Du Plessis et al., 2018)
${m_{T}}_{\max}$	Mortality rate per heat stress, °C/d	0.02	Assumes 10% daily mortality at 45 °C
$C_{\mathrm{wilting}}$	Critical wilting fraction	0.50	
$m_{\mathrm{wilting}}$	Mortality rate per desiccation stress, 1/d	0.2	Assumes 10% daily mortality for dry soil.

To generate an estimate of the location of suitable permanent FAW populations we took the annual mean intrinsic growth rates across months. This is a suitable measure of the permanence of a population as the sum of the intrinsic growth rate exponents in the exponential solution of $\frac{dN}{dt}$ represents the aggregated growth rate across a number of periods, n of duration t, i.e. $exp\left(r_{1}t\right)\times exp\left(r_{2}t\right)\;\times \;\ldots \;\times exp\left(r_{n}t\right)=exp\left((r_{1}+r_{2}+\ldots +r_{n})t\right)$ where $r_{i}$ is the intrinsic growth rate for the ith period. Dividing by the number of periods n (i.e., taking the mean) standardises the temporal units.

### 2.2 Short-range dispersal

Dispersal mostly occurs through winged FAW adults, although at smaller scales, dispersal to surrounding plants can occur *via* larvae (Garcia et al., 2018; 2016). Larval dispersal of both *Bt* resistant and susceptible strains of FAW exposed to *Bt* and non-*Bt* cotton under laboratory conditions, showed that neonate larvae can move 21.2 – 71.4 cm over a period of 12 h, although this was dependent on the host (Malaquias et al., 2017). However, longer distance dispersal in many neonate caterpillars is commonly achieved by ballooning, a process in which the neonate lowers itself on a strand of silk and is carried by the wind (Zalucki et al., 2002). This behaviour decreases as caterpillars age due to their increased weight (Zalucki et al., 2002). In a recent study, Sokame et al. (2020) found that, compared with other lepidopteran species, FAW neonate larvae were more successful at spreading to adjacent maize plants. Approximately 50% of FAW larvae exhibited ballooning behaviour, compared with approximately 30% in other species. Furthermore, FAW were able to colonise a mean of 9.8 of 10 maize plants placed at an 80-cm radius from an infested plant, suggesting a smaller proportion would likely be able to colonise plants at considerably further distances (Sokame et al., 2020).

In short-range dispersal, a simple location dispersal kernel specifying the probability density of an individual migrating from an origin cell (i) to a destination cell (j) during a timestep can be defined by a negative exponential function of distance between cells ($d_{i,j}$):

$$f_{i,j}=\exp\left(-\frac{d_{i,j}}{\alpha }\right)$$

where $d_{i,j}=\sqrt{{\left(x_{j}-x_{i}\right)}^{2}+{\left(y_{j}-y_{i}\right)}^{2}}$ (x and y represent cells in the horizonal and vertical directions, respectively) and α is a parameter that can be estimated from data where 2α is the mean dispersal distance (Nathan et al., 2012). This probability density can be truncated and discretised for a neighbourhood of cells ($S_{i}$) within a finite step distance ($d_{S}$) from cell i, where neighbourhood cell $s\in S_{i}|d_{i,n}<d_{S}$, assuming negligible short-distance migration beyond $d_{S}$. The discrete probability of an individual migrating from cell i to s is thus given by:

$$p_{i,s}=\frac{f_{i,s}}{\sum_{s}f_{i,s}}$$

We set the parameter α at 0.01, and step distance of $d_{S}$ of two cells or 18 km. This results in 1% of the population undertaking short-range dispersal to adjacent cells each time step, however, we found at large spatial scales, such as the North American continent, variation in this parameter did not have large impacts on populations dynamics, which was dominated by long-range dispersal and environmental suitability. Short-distance dispersal is likely to have greater implications at field scale simulations. Dispersal module parameters for short-distance dispersal (and other dispersal modules) are summarised in Table 2.

**Table 2:** Summary of dispersal module parameters, estimated values, and justification.

Module	Parameter description (units)	Estimated value	Justification
Short-distance dispersal	α*negative exponential coefficient (-)*	0.01	This parameter resulted in a 1% short-range dispersal rate to adjacent cells each time step. At large spatial scales, variation in this parameter did significantly impact populations dynamics, which were dominated by long-range dispersal and environmental suitability.
Long-distance dispersal	β*proportion of individuals that undergo long-distance dispersal (individuals/individual)*	0.05	This parameter allowed populations to reach Canada from its permanent range in the southern parts of the USA, as has been reported elsewhere, and is well within the maximum measured daily migration range of FAW.
Allee	θ*minimum viable population per grid cell (individuals)*	10 000	This parameter was somewhat arbitrarily assumed as it is inherently difficult to empirically quantify but was necessary to capture the process of mating after migration. Simulating variation around this parameter estimate did not cause large changes in seasonal spread predictions.

### 2.3 Long-range dispersal

**Fig. 1:**
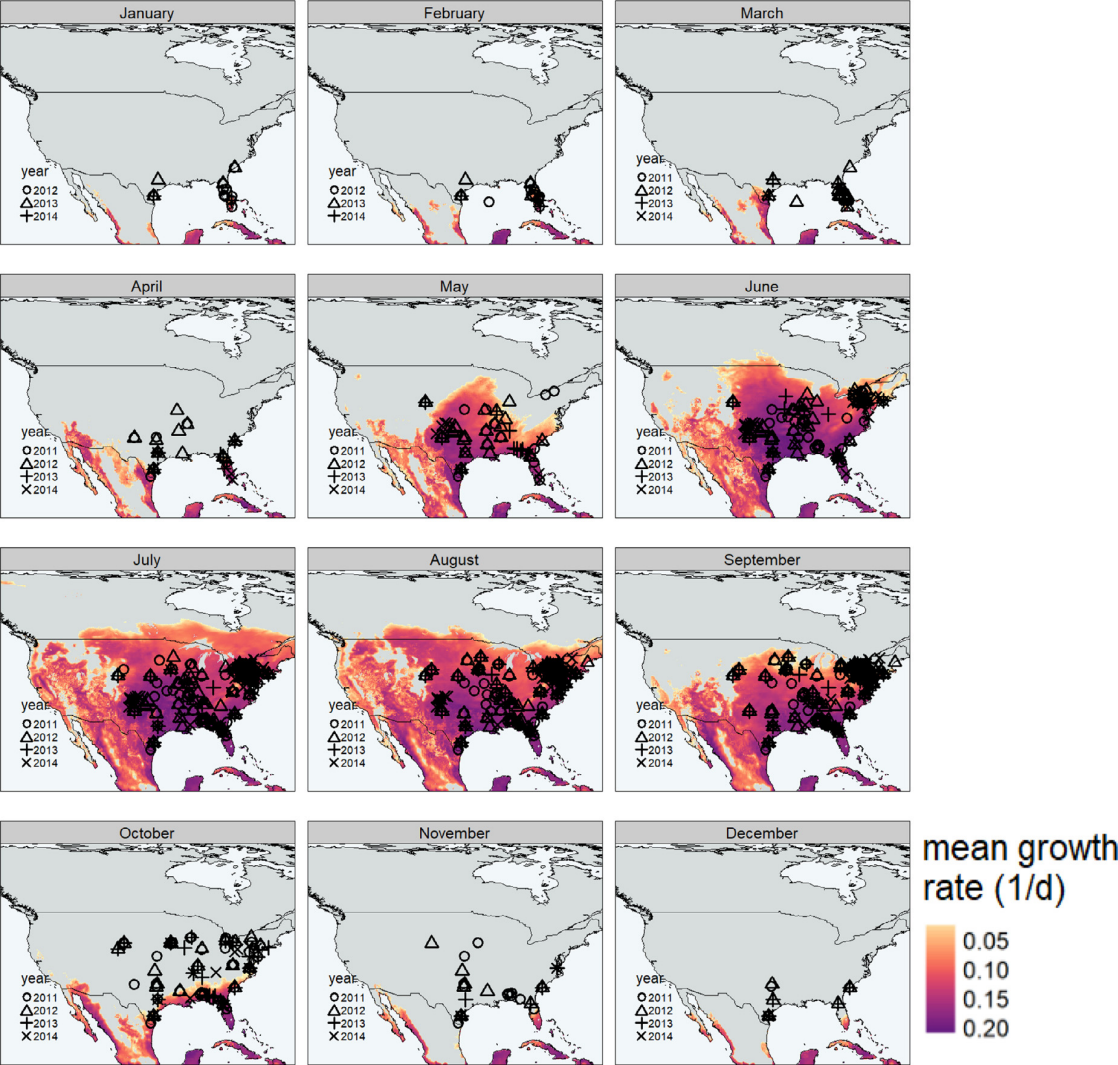
The monthly estimated population growth rate shown throughout the year for North America. Cold temperatures restrict permanent population persistence in cooler seasons. Symbols depict seasonal occurrence of FAW as demonstrated through time-stamped pheromone trapping records (Westbrook et al., 2016a).

Long-distance migration in moths is prevalent among many noctuid species, including FAW. In female moths, migration typically occurs early in the adult stage, before reproduction begins (Johnson 1969; 1960). In contrast, males exhibit more varied patterns of migration and reproduction (Johnson 1969). The most well studied example of the migration potential of FAW can be seen in the seasonal distribution of FAW across North America (Fig. 1). With the exception of southern Texas and Florida, which have relatively mild winters, FAW lacks the ability to overwinter in the USA (Foster and Cherry 1987). Therefore, the observed populations at higher (colder) latitudes are the result of annual movements of populations facilitated by increasing temperatures post-winter. Seasonal FAW populations have been observed as far north as Ontario, Canada (Rose et al., 1975), which represents an annual movement of over 2000 km from its permanent range (Wolf et al., 1990). Further, FAW has been observed to migrate over 400 km overnight (Wolf et al., 1990). Other noctuid moths can migrate much larger distances overnight (Chapman et al., 2010; Farrow 1984; Fox 1978).

Assumptions around long-distance dispersal can have profound impacts on predicted dispersal rates (Kot et al., 1996), but these dispersal rates are intrinsically difficult to quantify due to the large spatial scales at which they occur. While atmospheric transport processes have been convincingly implicated in the long-distance migration of FAW (Westbrook et al., 2016a) as well as other noctuid species (Wolf et al., 1990), here we opt for a simple long-distance dispersal module for increased computational tractability and to also test whether seasonal patterns can be explained if underlying climatic constraints are considered. The number of long-distance migrants moving from cell i, each timestep can be expressed as:

$$L_{i,R}=\beta N_{i}$$

Where R is the randomly selected destination within 540 km of cell i, β is the proportion migrating at each timestep, and $N_{i}$ is the population size at cell i. We assumed that in each grid cell with a FAW population, 5% of the population can disperse in a random direction to any grid cell at each weekly time step, which allowed populations to reach Canada from its permanent range in the southern parts of the USA, as has been reported (Rose et al., 1975). This reflects a dispersal distance of 90 km per night, well within the measured maximum migration range of FAW (Wolf et al., 1990).

### 2.4 Allee effects

Dispersing migrants may fail to colonise a vacant grid cell if populations are too small (density dependence). This can be captured by some critical population threshold, θ below which the grid cell's population will go extinct. To capture these Allee effects, we assume that 10 000 individuals per cell (or, on average, approximately 1 moth per ha) are required in each cell by the end of the time step, otherwise, all individuals in the cell become extinct. While this parameter is inherently difficult to empirically quantify (Rhainds 2010), variation around this parameter estimate did not cause large changes in seasonal spread predictions. Indeed, previous light trap studies found Noctuid moths *Mythimna convecta,* and *Helicoverpa* spp. were nearly all unmated, even at high densities, suggesting that the most mating occurs after migration when arriving populations are large (Coombs et al., 1993).

### 2.5 Gridded climatic data, simulations, and model evaluation

The Soil Moisture Active Passive (SMAP) data products derived from the SMAP satellite mission were used to estimate various climatic conditions relevant to habitat suitability (Entekhabi et al., 2010). In particular, we use SMAP Level 4 data products (version 4), which are model-derived value-added products that combine SMAP satellite observation with a land surface model and observation-based meteorological forcing data, including precipitation and temperature, to provide global gridded climatic and environmental data at the 9 km resolution (grid cell) every 3 h from April 2015 to present (Reichle et al., 2017). Two data fields were used to define climatic stressors including ‘surface_temp’: mean land surface temperature (K), and ‘land_fraction_wilting’: the fractional land area that is at wilting point based on soil moisture at 0–5 cm (m^3^ m^−3^) and estimated soil type.

Using gridded SMAP climatic data from 2015 to present and the parameters defined in Table 1, for each month, we calculated the mean daily stress limited growth rate: $r-\sum_{s}f\left(s,s_{c}\right)m_{s}$ using 3-hourly timesteps, which was then used to estimate intrinsic population growth across the world for each month. While carrying capacity *K* will vary based on climate and host-availability, for simplicity, we assumed a carrying capacity of 1 billion individuals per grid cell, or approximately 1 individual per m^2^, which reflects commonly observed field densities (Pair et al., 1991). Simulations were run at a 9-km grid cell resolution and weekly time steps using calculated monthly growth rates.

To validate model predictions, recent data on the global distribution was utilised (Wang et al., 2020). Following previous studies of FAW migration (Westbrook et al., 2016b; 2019), weekly monitored pheromone trap data from the USA national PestWatch database provided information on seasonal migration activity of FAW at numerous locations across the USA between 2011 and 2014.

To extend predictions to Australia to support pest preparedness activities, the mean annual population growth potential was estimated using climatic data from 2016 to 2019, which is expected to be associated with the location of permanent populations. Seasonal changes in suitability were estimated through monthly estimates of population growth potential using 2019 climatic data. To highlight the seasonal activity potential in key grain growing regions, population density through time was estimated from 100 replicate simulations using data from 2016 to 2019. This population activity potential was overlaid with the approximate growing seasons for Maize and Sorghum available at the state level (USDA 2020).

### 2.6 Data accessibility

Source code required to reproduce the analysis is available at the open-access GitHub repository https://github.com/cesaraustralia/FallArmyWorm

## 3 Results

The modelled monthly average population growth potential of FAW appears to correspond well with observed distributions under North American conditions (Fig. 1). Several unusual findings in the North American pheromone trapping data included a lack of population activity in northern areas in months predicted to be suitable for growth (e.g. in July), and the detection of moths in certain areas in months unsuitable for growth (e.g. in January).

**Fig. 2:**
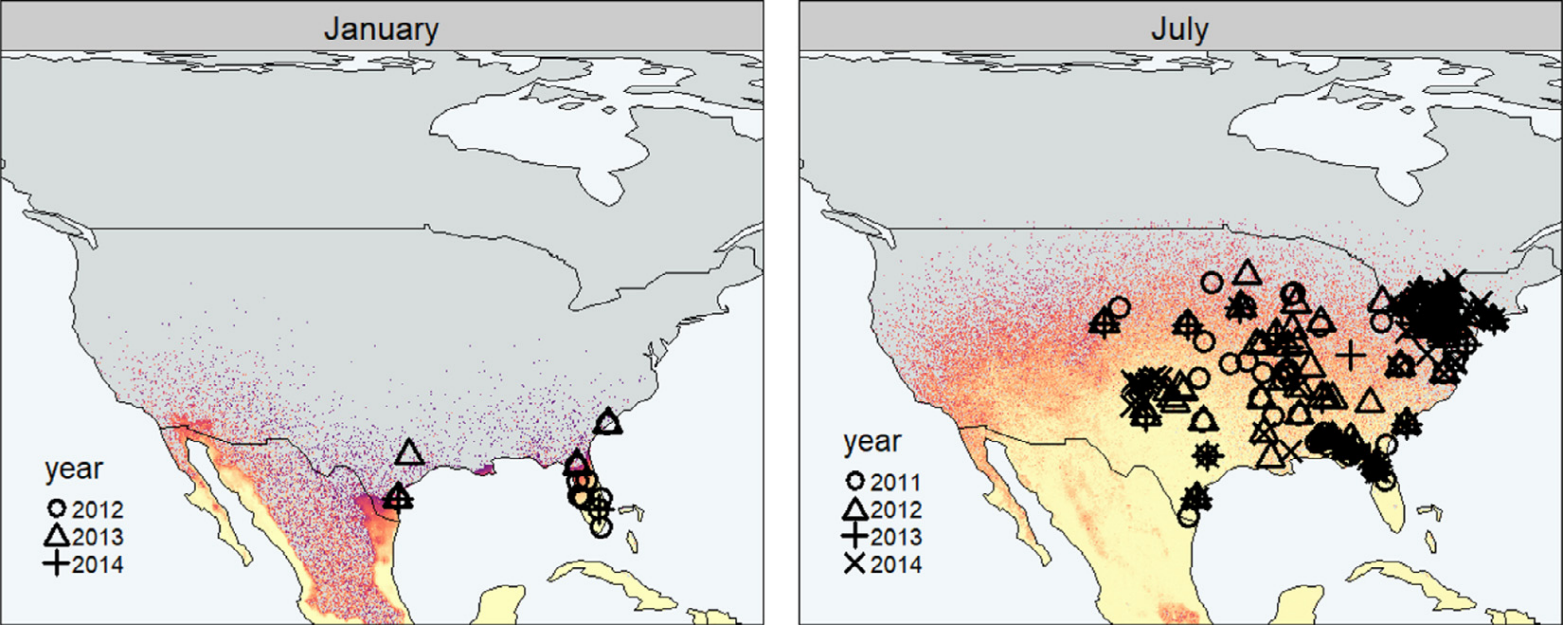
Dynamic growth potential layers combined with short-range and long-range dispersal processes allows the seasonal prediction of FAW dispersal potential in North America. Symbols depict seasonal occurrence of FAW in North America as demonstrated through time-stamped pheromone trapping records (Westbrook et al., 2016a). Predicted population density is denoted by the shading of the background with darker regions denoting smaller populations. The grey regions in the upper regions of the map denote areas where populations are estimated to be absent.

Incorporation of spread processes helps to explain these discrepancies. Indeed, a relatively simple model of dispersal processes was found to capture many of the seasonal dynamics of FAW in North America, which can be seen as static slices through time in Fig. 2. Thus, a simple dispersal model overlaid onto seasonal variability in population growth potential, at least qualitatively, captures the putative pathways of dispersal population dynamics of FAW in North America. This helps to explain the lack of populations in northern areas suitable for growth in July, and the detection of moths in January in areas predicted to be unsuitable for growth (Fig. 2).

**Fig. 3:**
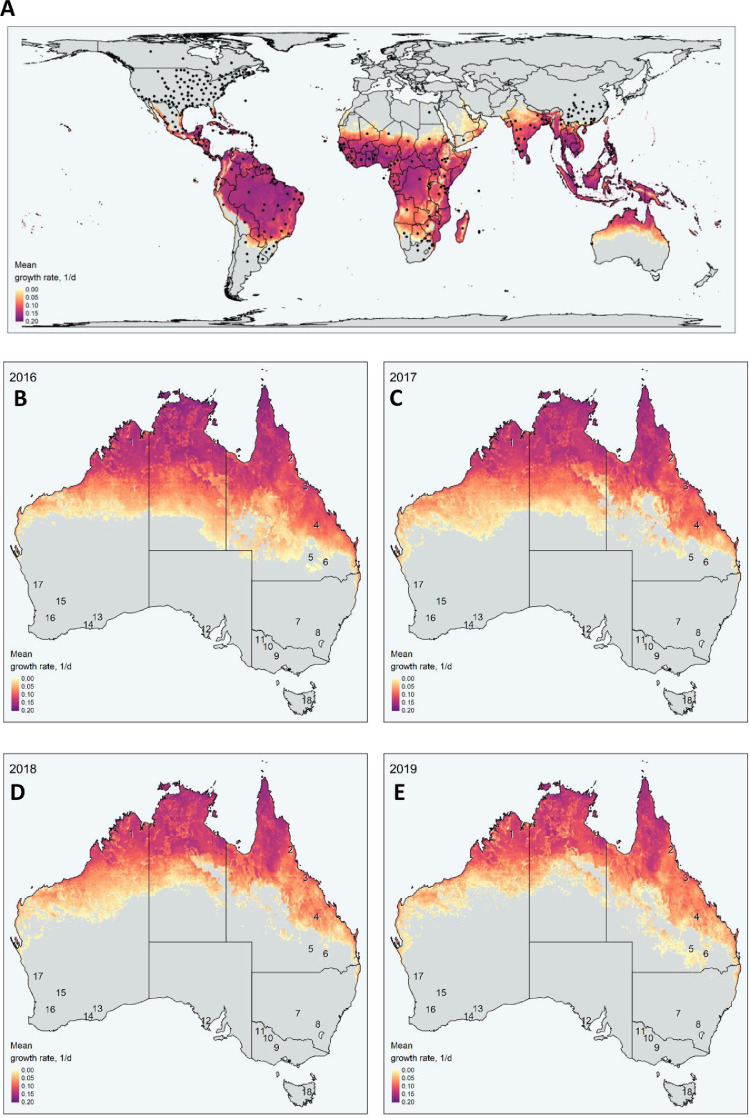
The annual estimated mean population growth rate of FAW representing areas predicted to support permanent populations across a global extent (**A**), and within Australia using climatic data from 2016 (**B**), 2017 (**C**), 2018 (**D**), 2019 (**E**), as denoted by the coloured legend. The grey regions of the map denote areas where estimated annual mean population growth rate is negative. Many known occurrence records across the world (grey circles) occur outside of this permanent range and highlight the importance of dispersal processes in the seasonal population dynamics of FAW. Numbers across Australia denote key grain growing regions as follows: 1 - WA Ord; 2 - QLD Atherton; 3 - QLD Burdekin; 4 - QLD Central; 5 - NSW North west - QLD South west; 6 - NSW North east - QLD South east; 7 - NSW Central; 8 - NSW VIC Slopes; 9 - VIC High Rainfall; 10 - SA VIC Bordertown - Wimmera; 11 - SA VIC Mallee; 12 - SA Midnorth - Lower Yorke Eyre; 13 - WA Mallee; 14 - WA Sandplain; 15 - WA Eastern; 16 - WA Central; 17 - WA Northern; 18 - TAS Grain.

The model was further validated by the global distribution of FAW (Fig. 3), with the majority of the known range captured by our model predictions. Importantly, many of the occurrence points fall in locations where the annual mean population growth potential is predicted to be below zero; indicating the proportion of FAW's range that can support permanent year-round populations and the seasonally suitable regions, such as the seasonally cool regions of North America. This highlights the importance of dispersal processes in forecasting the seasonal population dynamics of FAW.

When applied to Australia, we can explore the expected locations of permanent FAW populations; regions where the mean monthly population growth rate was estimated to be greater than zero (Fig. 3c-f). For key Australian grain growing regions, a time series of population activity is provided in Fig. 6. Current estimates suggest that the window of activity is longer for more northern growing regions (see Fig. 3). Some locations, such as Region 1 - WA Ord and Region 3 - QLD Burdekin, will likely see year-round populations. Relatively cooler, more southern regions, such as Region 16 - WA Central and Region 11 - SA VIC Mallee, will see the greatest population build up following summer, with some potential for spring population outbreaks. Region 9 - VIC High Rainfall region will potentially see migration commence from October, with populations building up in late summer and into autumn. While the model uncertainty increases significantly towards the expected southern limits of its range, given the cold climate of Region 18 - TAS Grain, there is a low likelihood of FAW large populations being observed.

The seasonal timing of FAW migration and population build-up was further compared with the growing period of maize and sorghum within each region of Australia (Fig. 5, data from USDA 2020). For regions into which FAW must first migrate before establishing populations (e.g. Region 17 - WA Central, Region 7 - NSW Central and Region 11 - SA Vic Mallee), the timing of adult arrival is predicted to occur shortly after crop emergence in spring. This suggests that population build-up of FAW in these regions will not be limited by mismatches in the timing of these crop hosts.

## 4 Discussion

Using a spatial model of population growth and spread potential informed by existing biological and climatic data, we simulated seasonal population activity potential of FAW, with a focus on Australia's grain production regions. Our results show that, in Australia, the large spread potential of FAW will allow it to exploit temporarily favourable conditions for population growth across highly variable climatic conditions. It is estimated that FAW populations would be present in a wide range of grain growing regions at certain times of year, but importantly, the expected seasonal activity will vary markedly between regions and years depending on climatic conditions. The window of activity for FAW will be longer for growing regions further north, with some regions possessing conditions conducive to year-round population survival. Seasonal migrations from this permanent range into southern regions, where large areas of annual grain crops are grown annually, are predicted to commence from October, i.e. spring, with populations subsequently building up into summer.

In comparison to previous modelling approaches that use long-term (annual) climate averages (Du Plessis et al., 2018), our FAW model used daily climatic data which allowed the estimation of seasonal variation in population growth potential. The incorporation of population growth and dispersal processes facilitated the estimation of seasonal activity potential through migrating and growing populations. While our simple dispersal model coupled with estimated weekly population growth rates could explain broad patterns in the observations of the seasonal dynamics of FAW, the role of atmospheric conditions on the long-range dispersal and deposition of FAW has also been shown to increase predictability (Westbrook and Sparks 1986; Mitchell et al., 1991; Westbrook 2008; Westbrook et al., 2016a; 2019). Some of the most sophisticated attempts to model the seasonal migrations of FAW are represented by Westbrook et al. (2016b; 2019), in which a simple crop and pest phenology model was coupled with spatial layers on commodity production and the USA National Oceanic and Atmospheric Administration's Hybrid Single Particle Lagrangian Integrated Trajectory Model (HYSPLIT) (Draxler and Hess 1998). This allowed for the incorporation of complex atmospheric processes that translocate and deposit airborne particles, and also for the prediction of regional variation in FAW moth trap activity. Generally, most studies on the dispersal potential of FAW have been conducted in North America, where permanent populations in southern Texas and southern Florida disperse northward following winter. Data from widely distributed pheromone traps has been used to estimate likely spread patterns of FAW in several studies (Nagoshi and Meagher 2004a; Meagher and Nagoshi 2004; Westbrook et al., 2016a). There will be a need for the incorporation of additional field observations as FAW continues to be studied outside of its native range. This could be expediated through crop monitoring support tools and compilation of field scouting efforts, such as FAO's FAMEWS global platform (www.fao.org/fall-armyworm/monitoring-tools/famews-global-platform/). Such programs demonstrate the limitations of using pheromone trap catches to validate these models, as there is uncertainty surrounding the relationship between trap catches and observed rates of plant infestation in the field. The FAMEWS data includes frequent observations of high field infestation rates of FAW without trap catches (or vice versa) (Supp. Fig. S4). Nevertheless, a general positive relationship exists between trap catches and observed field infestation rates.

A diverse range of atmospheric processes are known to facilitate insect transport (Drake and Farrow 1988). In China, atmospheric transport processes are more predictable where prevailing winds (or sea breezes) drive patterns of atmospheric transport of insects (Li et al., 2020). In Australia, with a few exceptions (e.g., the south-east trade winds affecting coastal northern Queensland or the winter westerlies of southern Australia), there is a lack of seasonal prevailing winds (Gregg et al., 2001). Instead, Australia typically sees sporadic and short-lived winds that are favourable for migration (i.e., sufficiently strong, and warm). Especially in spring, but also in summer, these are often northerlies or north-westerlies, ahead of cold fronts. In summer, there are also the post-frontal south-westerlies. Thus, in Australia, long-range wind-borne movement is a more stochastic process, depending on migration-ready populations, local weather conducive to nocturnal take-off and climbing to high altitudes, and atmospheric transport mechanisms capable of carrying FAW across long-distances. In the southern half of Australia in spring, these coincidences are often associated with the passage of cold fronts, and the window of opportunity may be as short as a week (Drake 1994). The presence or absence of these coincidences can determine whether, in any given season, moths will be found outside the range predicted by dispersal models such as ours, or conversely, not found in locations where the model predicts they should be. It will be important to determine whether FAW can exploit the same opportunities for long range movement that endemic species such as *H. punctigera* utilise (Drake 1994; Gregg et al., 2001).

Our work indicates FAW will persist in the northern regions of Australia year-round, while its ability to disperse over long distances is likely to allow this pest to exploit seasonally favourable conditions for population growth in other areas. FAW will almost certainly be observed in a wide range of crop production regions in Australia, but importantly, the expected seasonal activity will vary markedly between regions, which in turn will require region-specific management strategies to be developed. In northern growing regions, the permanent range of FAW is expected to retract and expand based on inter-annual climatic variability and changes to suitability. More southern regions will see migrating FAW populations generally from October, with population densities increasing into summer. Cold climates are expected to regulate the distribution of FAW in the southernmost regions of Australia.

### 4.1 Implications for pest management

Our predictions for the seasonal activity potential of FAW in Australia will aid in targeting regional preparedness activities and research, particularly when overlaid with high volume production regions of vulnerable crops. Sorghum is the main summer grain crop grown in Australia (ABARES 2020) and has already been extensively attacked by FAW since its invasion in February 2020; thus constituting a key susceptible host crop. Maize is a minor summer crop; however, it is very attractive to FAW (Meagher et al., 2004) and is also expected to be impacted throughout the summer period. Maize production is largely distributed in eastern Australia, with most production occurring in southern Queensland and New South Wales (Supp. Fig. S3). Sorghum production is mainly aggregated along the eastern coast Australia, particularly in Queensland and northern New South Wales, although there are small pockets of production in other states (Supp. Fig. S3). These geographic regions overlap with areas into which FAW could disperse and persist over summer, and so regular monitoring of these crops will be important in the future.

Our findings suggest that on the basis on seasonal distribution patterns of FAW, winter crops grown in New South Wales, Victoria and South Australia are at comparatively lower risk. However, given the inter-annual variability in climate in these regions, and associated variation in crop sowing dates and FAW migration, some regions may nonetheless be impacted in some years. For example, if FAW migrated into central NSW as predicted, populations could persist with high population growth well into autumn. Winter crops (e.g. pulses such as chickpeas and lentils) are routinely sown in early-mid autumn in these regions (NSW DPI 2020), thus FAW may end up migrating into these fields and cause considerable vegetative damage to emerging seedlings before winter temperatures constrain their development. Continued monitoring of these dynamics is required to attain a more complete understanding of the risk FAW poses to crops in southern regions of its potential range. For commodities with little data on impact potential (e.g. winter cereals and legumes), the alignment of crop phenology and expected seasonal activity offers an efficient, though coarse, method to identify at-risk commodities.

**Fig. 4:**
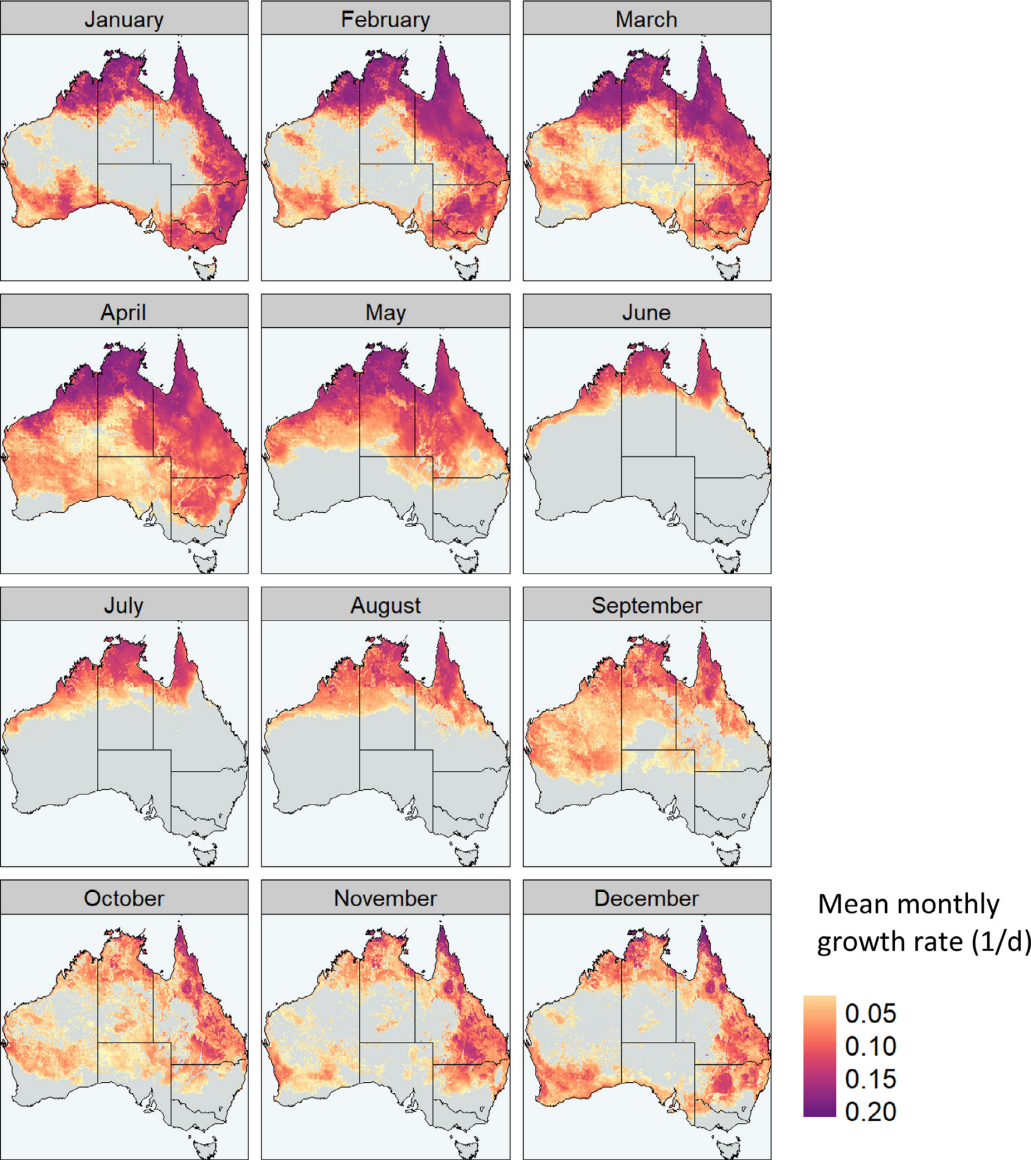
The monthly estimated population growth rate in Australia shown throughout the year of 2019. Hot and cool temperatures, as well as dry conditions restrict permanent population persistence.

**Fig. 5:**
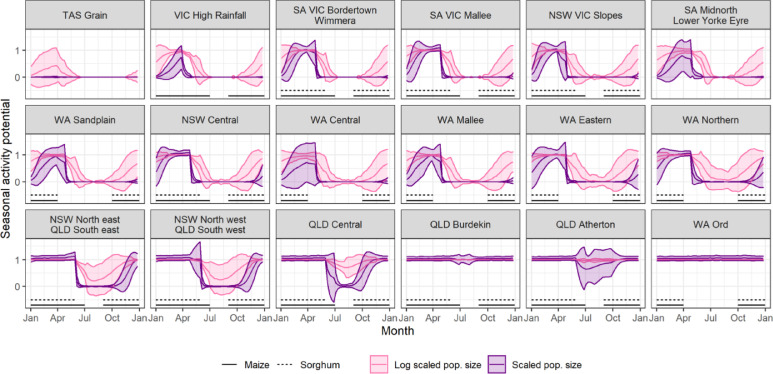
The predicted seasonal activity potential for key grain growing regions in Australia ordered by decreasing latitude. Locations of regions are shown in Fig. 3. Higher spread and infestation potential indicate higher FAW activity, with the vertical width of the shaded region denoting increased uncertainty. To visualise both small populations (associated with incoming migrants) and larger populations (associated with build-up), population size and log-transformed population size both scaled to unity are presented. Approximate growing seasons for Maize and Sorghum available at the state level (USDA 2020) are overlaid as horizontal lines.

Coupling the predicted changes in suitability measured through time in Fig. 4 and population activity in Fig. 5, offers a method to determine approximate pathways of seasonal FAW dispersal into southern cropping regions following winter. For example, migrations into south Australia in spring are likely to originate from eastern and north-eastern Australia, rather than central Australia or Western Australia as evidenced by changing patterns of predicted suitability. However, the large inland areas of eastern Australia, with high population growth potential from October to November, include significant areas of native vegetation. Thus, a key knowledge gap in Australia will be the suitability of native grass species for FAW and the contribution of inland native vegetation to source populations of migrating FAW.

### 4.2 Future work

Future predictive work may incorporate real-time climatic data, which could facilitate forecasting potential of seasonal risk ahead of time. This will first require validation against field observations that are not yet available for Australia at this early stage of the pest's establishment. Adjustment of biological parameters in the model can be used to identify sensitive FAW traits that may lead to increased impact (e.g. in the event of future incursions or evolution of new FAW strains). While FAW has been declared established in Australia, there will be a continued need to consider pathways of overseas populations of FAW migrating into Australia due to the presence of unique biotypes. These biotypes can have important management implications through the introduction of novel pesticide resistance mechanisms that impede control (Umina et al., 2019), different host preferences and associated crop damage (Nagoshi and Meagher 2004b), or novel traits that enhance establishment in Australian environmental conditions (Weeks et al., 2002). While our study only considered climatic responses of FAW at the species level, variation among FAW strains and local evolution may prove important for finer scaled predictive applications.

The prediction of FAW outbreaks will also require further investigation. In the USA, the probability of a general invasion of FAW is largely dependent upon the prevailing climatic conditions during the winter months in the species’ permanent range (Luginbill 1928). It has been noted that, within this permanent range, FAW thrives when periods of cooler temperatures coincide with relatively higher rainfall, which, in addition to causing an abundance of host plants (Westbrook and Sparks 1986), can also lead to a deficit in natural enemies (Hogg et al., 1982). Thus, without the top-down control that would otherwise be exerted by natural enemies, high FAW population growth can occur. By the time conditions become favourable for natural enemies, FAW tends to migrate northward and invade northerly regions of the USA. Pair et al. (1986) examined the seasonal distribution of parasitoids and found that an important species, *Chelonus insularis*, followed FAW north during such migrations but lagged significantly in timing. However, Hogg et al. (1982) found high parasitism rates in areas where FAW migrate. The incorporation of parasitoids (and other natural enemies) and associated FAW mortality will be an important consideration in future models that aim to increase the predictability of outbreaks in Australia. The modelling framework we have developed for FAW will enable us to incorporate additional biological complexity, such as interactions with natural enemies, which will help to consolidate our ecological understanding and improve our capacity to predict outbreaks.

### 4.3 Conclusion

Despite some uncertainties, this study provides much needed insights into the potential distribution and phenology of FAW across Australia. The modelling approach adopted allows us to tease apart geographic regions into which FAW may disperse but not establish, in comparison to those in which permanent, year-round populations are likely to persist. Moreover, the high temporal (weekly) resolution of the model outputs forecast inter-annual and seasonal variation in FAW's population dynamics. These predictions can allow farmers and farm advisors to tailor their strategies for monitoring and managing FAW. While the model will need to be updated and further validated as more information of the pest in Australia is collected, the modelling framework developed here provides a sound baseline with which the ongoing invasion of FAW can be monitored and, where possible, managed. Further, our models will provide a useful framework for other countries should FAW invade in the future.

## Declaration of Competing Interest

The authors declare that they have no known competing financial interests or personal relationships that could have appeared to influence the work reported in this paper.

## Appendix Supplementary materials

Image, application 1
