# Supplementary material for: Regional and seasonal activity predictions for fall armyworm in Australia
Source: Curr Res Insect Sci. 2021 Jan 23;1:100010. doi: 10.1016/j.cris.2021.100010 (PMC9387490; doi:10.1016/j.cris.2021.100010)
Supplement: Supplementary file 1 [file mmc1.docx]

Maino et al.

Regional and seasonal activity predictions for fall armyworm in Australia

**Supporting Information**


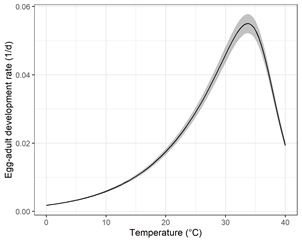

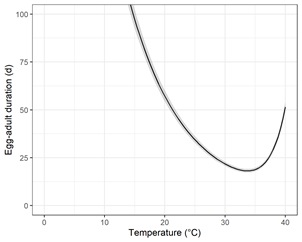


**Figure S1.** The temperature response of FAW egg-adult development (left panel) and duration (right panel) estimated from constant and variable temperature regimes Barfield et al. (1978) with the shaded region denoting the standard error.


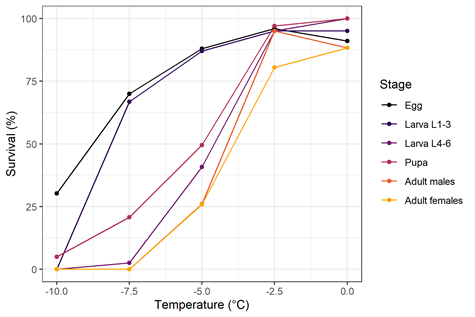


**Figure S2**. Survival of cool-acclimated life stages of FAW following 3-h cold exposure at various constant temperatures as measured by Foster and Cherry (1987).

**
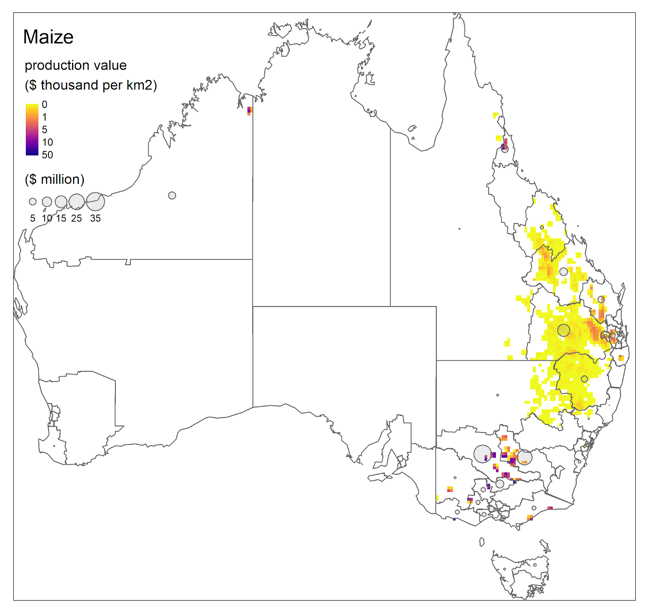

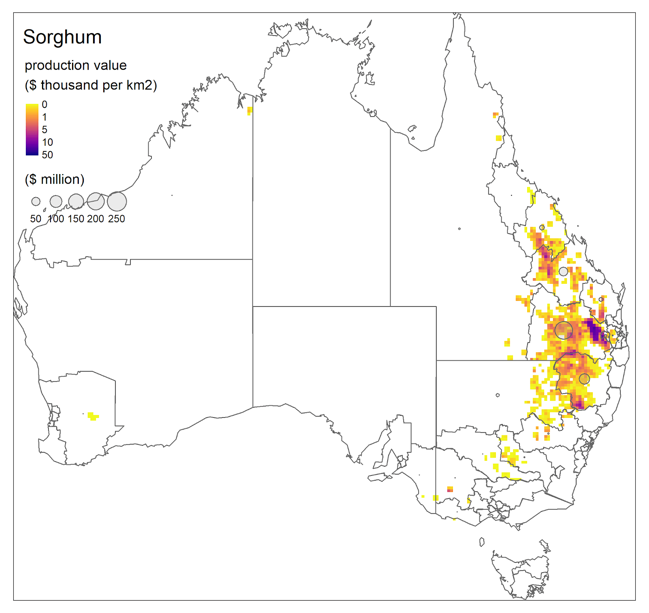
**

**Figure S3.** Maize and sorghum production value (AU$ thousand per km^2^) throughout Australia. Increases in value are represented from light (AU$0 per km^2^) to dark (AU$50,000 per km^2^). Total production values for each statistical area are denoted by the size of the circles at the centre of the statistical region. Data compiled from the Australian Bureau of Statistics 2017-18.

##
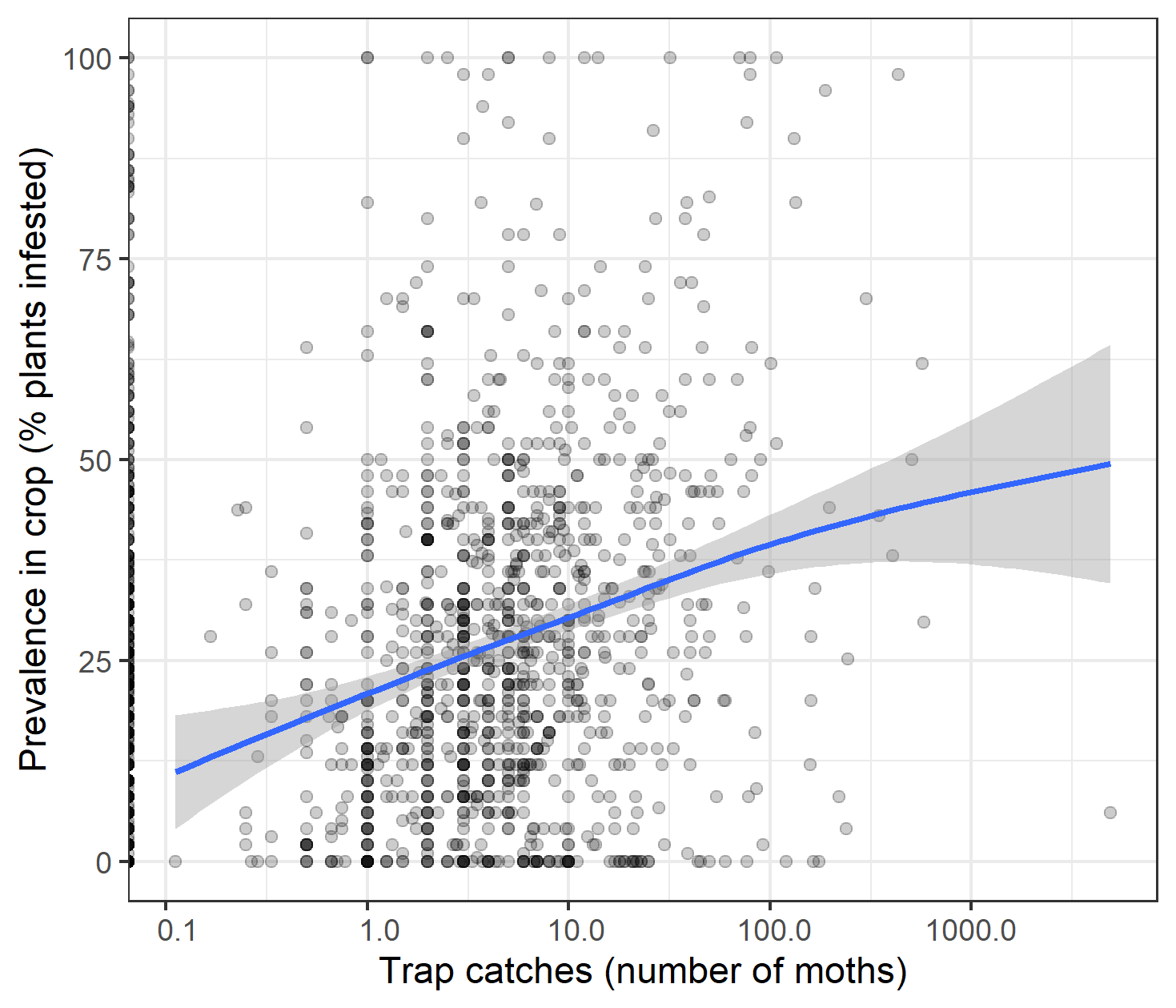


**Figure S4.** FAO’s FAMEWS platform provides an international crop monitoring tool comprising FAW trap catches and field scouting data, which we have used to explore the relationship between monthly trap catches and corresponding observed field infestations. The majority of data consists of observations on maize. Countries represented in the dataset include Bangladesh, Benin, Botswana, Burkina Faso, Burundi, Cape Verde, Central African Republic, East Timor, Egypt, Eswatini, Ethiopia, Ghana, Guinea, Guinea-Bissau, India, Iran (Islamic Republic of), Italy, Ivory Coast, Kenya, Liberia, Madagascar, Malawi, Mali, Mozambique, Myanmar, Nigeria, Russia, Rwanda, Senegal, Somalia, South Africa, South Sudan, Sudan, Thailand, Togo, Uganda, United Arab Emirates, United Republic of Tanzania, Yemen, Zambia, and Zimbabwe. Data was kindly provided by FAO’s Fabio Lana. The FAMEWS platform is available at http://www.fao.org/fall-armyworm/monitoring-tools/famews-global-platform/en/

**References**

Barfield, C. S., E. R. Mitchell, and S. L. Poe. 1978. “A Temperature-Dependent Model for Fall Armyworm Development.” *Ann. Entomol. Soc. Am.* 71: 70–74.

Foster, R E, and R H Cherry. 1987. “Survival of Fall Armyworm, Spodoptera Frugiperda,(Lepidoptera: Noctuidae) Exposed to Cold Temperatures.” *Florida Entomologist*, 419–22.
